# Association of preterm birth with severity of molecularly-confirmed acute viral respiratory illness presenting to the emergency department: a multi-year analysis

**DOI:** 10.3389/fped.2026.1841637

**Published:** 2026-06-02

**Authors:** Rashed A. Hasan, Brian M. Nolan

**Affiliations:** 1 Department of Pediatrics, Hurley Medical Center, Flint, MI, United States; 2 Department of Pediatrics, Michigan State University, East Lansing, MI, United States

**Keywords:** acute viral respiratory illness, hospital length of stay (LOS), preterm (birth), respiratory support, respiratory syncytial virus (RSV)

## Abstract

**Background:**

Prematurity is a recognized risk factor for respiratory morbidity, but its association with illness severity across viral etiologies in contemporary emergency department (ED) cohorts is not well studied.

**Objective:**

To evaluate the association between prematurity and the severity of molecularly-confirmed acute viral respiratory illness (AVRI) in children presenting to the ED.

**Methods:**

We conducted a retrospective cohort study of pediatric ED encounters from January 1, 2019, through December 31, 2025. Children 0 -18 years of age who presented to the ED with clinically significant AVRI defined as respiratory distress with hypoxemia, or hypercarbia, and who underwent respiratory viral PCR testing with at least one pathogen identified were included in the study. Prematurity was defined as birth before 37 completed weeks of gestation. Outcomes included respiratory support modalities requirement, ICU admission, hospital LOS, and mortality. Multivariable logistic regression analysis was used for binary outcomes, and generalized linear model with gamma distribution and log link was used for LOS, adjusting for age, sex, year, and viral agents.

**Results:**

A total of 16,518 met inclusion criteria, of which 2,384 encounters were born preterm while 14,134 encounters were born at term. Preterm encounters were younger (21 months vs. 26 months). The most frequently detected pathogens were rhinovirus and/or enterovirus (RV/EV) in 6,432 encounters (38.9%), RSV in 2,427 (14.7%), adenovirus in 1,523 (9.2%), parainfluenza in 1,315 (8.0%), influenza in 858 (5.2%), and human metapneumovirus in 600 (3.6%). Coinfection occurred in 12.4% of encounters and was more common among preterm encounters (14.3% vs. 12.1%, *p* = 0.003). Preterm children more frequently required supplemental oxygen (39.0% vs. 30.2%), HFNC (23.8% vs. 16.4%0, noninvasive positive pressure ventilation (13.0% vs. 8.6%), and invasive mechanical ventilation (4.3% vs. 2.0%) (all *p* < 0.001). In adjusted analysis, prematurity was independently associated with higher odds of PICU admission (aOR, 1.57;95% CI, 1.43 to 1.72; *p* < 0.001) and approximately 17% longer hospital LOS. Mortality was rare (0.09%) and did not differ between groups.

**Conclusions:**

Prematurity was associated with higher respiratory support requirement, greater likelihood of PICU admission and longer hospital LOS in children presenting to the ED with clinically significant AVRI.

## Introduction

Acute viral respiratory illness (AVRI) is a major cause of emergency department (ED) visits and hospitalization in children (1). Most published data on severe viral respiratory illness have focused on the respiratory syncytial virus (RSV), which has historically been the leading cause of ED visits and hospitalizations for acute viral respiratory disease, particularly in infants and young children (2–5). However, pediatric ARVI is caused by a diverse group of viruses, including rhinovirus and/or enterovirus (RV/EV), RSV, adenovirus, influenza, parainfluenza, and human metapneumovirus (hMPV) (1). RV/EV has increasingly been recognized as an important cause of AVRI requiring ED visits and hospitalization and continued to circulate during the COVID-19 pandemic even when other respiratory viruses declined (6–9).

Prematurity is a well-recognized risk factor for morbidity and mortality in the setting of AVRI, related to impaired lung development, limited pulmonary reserve, and altered immune function (10, 11). Infants and children with history of preterm birth are more susceptible to lower respiratory tract disease and are more likely to develop severe illness requiring hospitalization and advanced respiratory support (12–14). Although prior studies have evaluated morbidity associated with specific viruses, fewer data have examined independent contribution of prematurity to illness severity across multiple viral etiologies within a single contemporary pediatric cohort (2–5). The objectives of the current study were to evaluate the association between prematurity and severity of molecularly-confirmed AVRI among children presenting to the ED. Our hypothesis was that prematurity would be independently associated with more severe illness, as measured by greater respiratory support requirements, higher likelihood of pediatric intensive care unit (PICU) admission, longer hospital LOS, and higher mortality.

## Methods

### Study design, setting and population

We conducted a retrospective cohort study of pediatric ED encounters at a tertiary care hospital from January 1, 2019, through December 31, 2025. The ED has approximately 90,000 visits annually, including one-third pediatric visits. We defined a clinically significant AVRI as the presence of respiratory distress, hypoxemia or hypercapnia to capture clinically meaningful AVRI presentation in the ED, although it may not isolate lower respiratory tract disease with complete specificity. These patients underwent reverse transcriptase polymerase chain reaction (PCR) testing on a respiratory sample (Biofire Respiratory panel 2.1, BioMerieux, Salt Lake city, UT) which detects the following pathogens: RV/EV, RSV, adenovirus, influenza A &B, parainfluenza 1-4, human metapneumovirus (hMPV), SARS-COV-2, and seasonal coronaviruses (sCOVs) 220 E, HKU1, NL63, OC43, mycoplasma and chlamydia.

The study cohort was derived from all pediatric encounters 0–18 yeas, presenting to the ED with significant AVRI and at least one pathogen identified on the respiratory PCR test. Children with incomplete data for analysis were excluded. The institutional review board approved the study (IRB # 23332340-2) and waived the need for consent.

### Data collection

Demographics, clinical, and virological data were extracted from the electronic health records (EHR).

### Exposure

The primary exposure was preterm birth, defined as documentation of birth before 37 completed weeks of gestation.

### Outcomes

The primary outcomes included:

1. Respiratory support: including oxygen therapy of any type, advanced respiratory support measures including, high-flow humidified nasal cannula (HFNC), non-invasive continuous/bilevel positive pressure ventilation (CPAP/BPAP) and tracheal intubation with positive pressure ventilation (mechanical ventilation) overall and stratified by gestational age.
2. Admission to pediatric intensive care unit (PICU)
3. Hospital length of stay (LOS), and
4. Mortality

### Covariates

Severity of illness at the index encounter was divided into three categories: mild (ED visit only without hospital admission), moderate (admission to a pediatric ward), and severe (admission to intensive care unit). Additional covariates included age, sex, calendar year, viral pathogen, seasonality of testing, ED resource utilization, and intensity of viral testing. Testing intensity was defined as the number of respiratory panels obtained within 48 h of the initial ED arrival.

### Statistical analysis

Data are presented as median with IQR or mean with standard deviation for continuous data and counts with percentages for categorical data as appropriate. Multivariable logistic regression analysis was used for binary outcomes, including PICU outcome and other dichotomous severity outcomes. Hospital length of stay (LOS) was analyzed using a generalized linear model with gamma distribution and log link. Models were adjusted for age, sex, calendar year, and viral pathogen. Because some patients contributed more than one encounter and analyses were conducted at the encounter level, all inferential models accounted for within-patient correlation.

To evaluate heterogeneity within the preterm population, preterm birth was stratified by gestational age (GA) categories (32 to 36 weeks, 28 to 31 weeks, and < 28 weeks) with term infants serving as the reference group. Multivariable models were used to estimate the association between GA strata and severe outcomes, including the need for advanced respiratory support. Additional analyses incorporated prior encounters as a proxy for prior disease burden, and virus-specific models were performed to assess whether the association between prematurity and severity differed by viral pathogen. Interaction terms between prematurity and viral pathogen were also examined. Fisher exact testing was used to compare mortality because death was rare.

Statistical analyses were performed using SPSS V 31 (IBM, Armonk, NY) and Posit (Version 4.2.2), with the survival, MASS and tidyverse packages (Version 1.3.2), pandas version 1.5.0 for data cleaning and inferential statistics using Scipy library version 1.9.0 (Posit PBC, Boston, MA). Statistical significance was set as two-sided with a *p* < 0.05 as significant.

This study is reported in accordance with the STROBE guidelines for observation studies.

## Results

### Demographic and clinical characteristics

Among 16,518 encounters from 2019 to 2025 included in the analytic cohort, 2,384 encounters had history of preterm birth, and 14,134 encounters were born at term. Table 1 presents, demographic and clinical characteristics of the cohort. Preterm children were younger than term children (median age 21 months [IQR 8–56] vs. 28 months [IQR 10–74], *p* < 0.001). Sex distribution did not differ between groups (*p* = 0.6).

**Table 1:** Characteristics of children with history of preterm birth compared with age-matched non-preterm controls (2019–2025).

Characteristic	Overall (*N* = 16,518)*	Term (*N* = 14,134)*	Preterm (*N* = 2,384)*	*P* value†
Age, months	26.0 (9.0, 71.0)	28.0 (10.0, 74.0)	21.0 (8.0, 56.0)	<0.001
Sex				0.6
Female	7,527 (46%)	6,454 (46%)	1,073 (45%)	
Male	8,991 (54%)	7,680 (54%)	1,311 (55%)	
Year				<0.001
2019	1,729 (10%)	1,386 (9.8%)	343 (14%)	
2020	1,303 (7.9%)	1,101 (7.8%)	202 (8.5%)	
2021	3,298 (20%)	2,881 (20%)	417 (17%)	
2022	2,791 (17%)	2,386 (17%)	405 (17%)	
2023	1,959 (12%)	1,669 (12%)	290 (12%)	
2024	2,718 (16%)	2,326 (16%)	392 (16%)	
2025	2,720 (16%)	2,385 (17%)	335 (14%)	
PICU admission	5,599 (34%)	4,583 (32%)	1,016 (43%)	<0.001
Length of stay, days	1.0 (0.0, 3.0)	1.0 (0.0, 3.0)	2.0 (0.0, 4.0)	<0.001

* Data are presented as median (Q1, Q3) or *n* (%).

† Wilcoxon rank-sum test, Fisher exact test, or Pearson chi-square test, as appropriate.

The most frequently detected pathogens were RV/EV in 6,432.0 encounters (38.9%), RSV in 2,427.0 (14.7%), adenovirus in 1,523.0 (9.2%), Parainfluenza in 1,315.0 (8.0%), influenza 858.0 (5.2%), and hMPV 600.0 (3.6%). Viral coinfection, defined as detection of two or more viruses during the same encounter, were identified in 12.4% of encounters (2,053/16,518) overall and was more common among preterm children (344 of 2,384 [14.3%] vs. 1,712 of 14,134 [ 12.1%], *p* = 0.003).

### Temporal patterns

**Figure 1:**
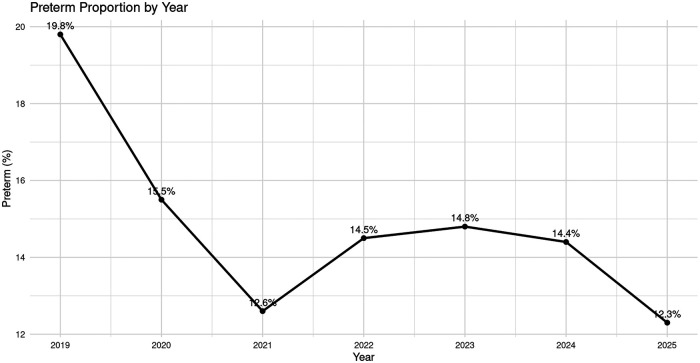
The proportion of emergency department encounters with history of preterm birth across the study years 2019 through 2025.

**Figure 2:**
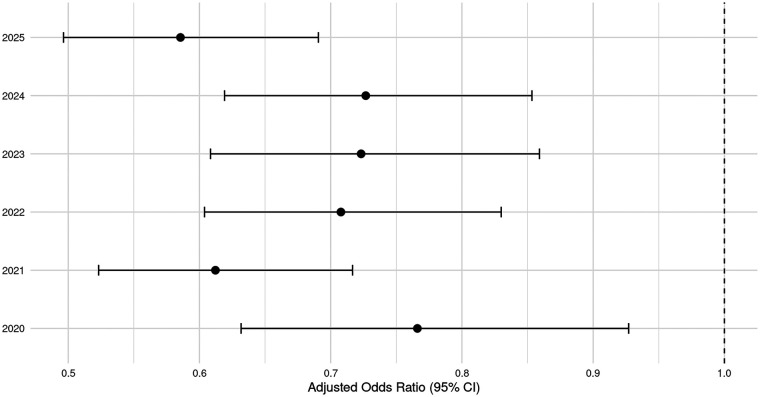
The odds of history of preterm birth in emergency department encounters in 2020 through 2025 compared to 2019. Adjusted odds ratio (aOR) with 95% confidence intervals (95%CI): the dots are the estimates, and the horizontal lines are the 95% CI. The vertical dashed line represents the point of no difference (null).

**Figure 3:**
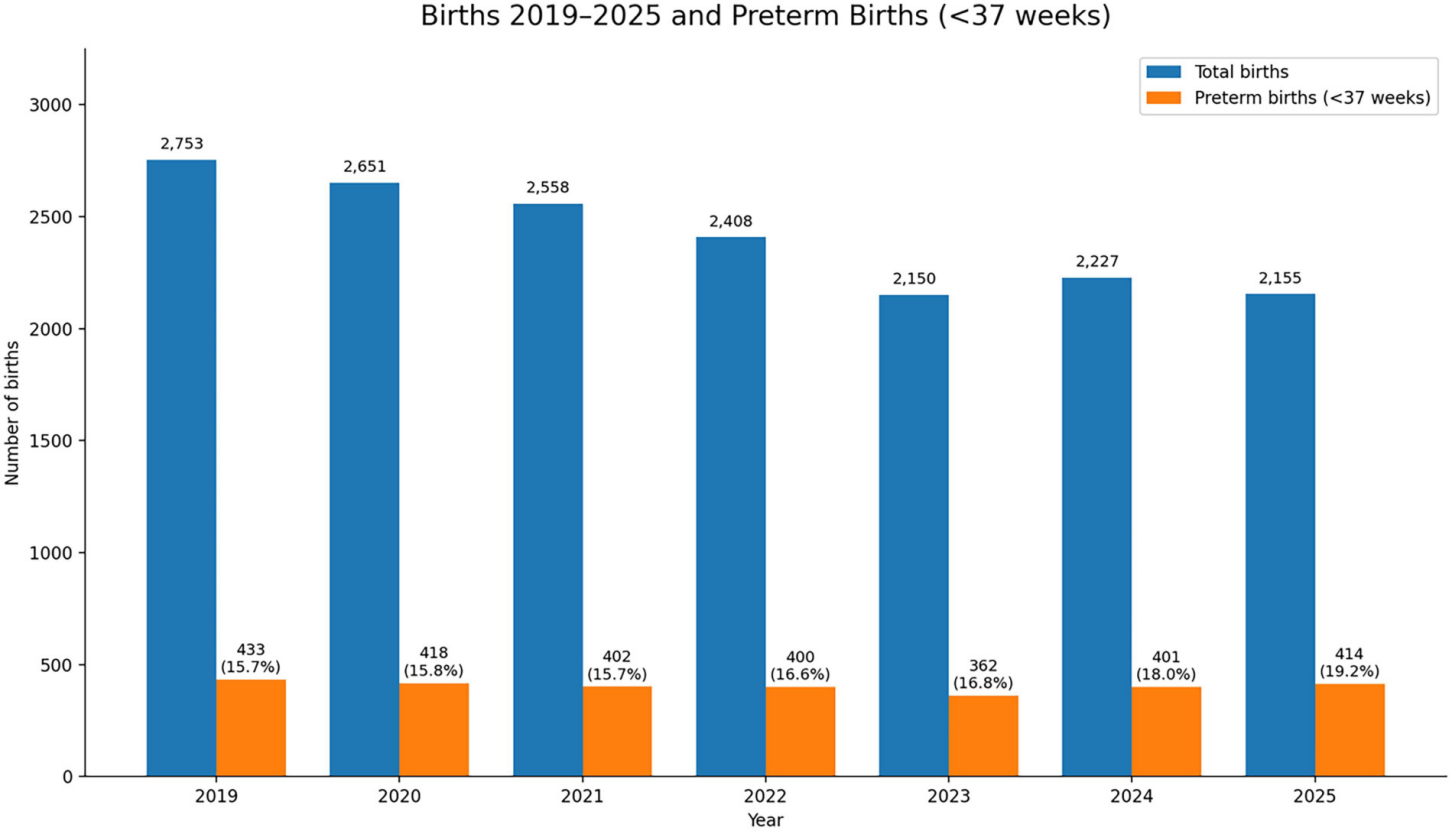
The proportion of institutional preterm birth across the study years 2019 through 2025.

The proportion of encounters with history of preterm birth declined over the period of the study, from 19.8% in 2019 to 12.3% in 2025 (Figure 1). Compared with 2019, the odds of an encounter involving a child with history of preterm birth were lower in each subsequent year in unadjusted analyses. After adjustment for age and sex, the odds remained significantly lower from 2020 through 2025, with the largest reductions observed in 2021 (adjusted odds ratio [aOR], 0.61; 95%, 0.51 to 0.72) and 2025 (aOR, 0.59; 95% CI, 0.50 to 0.69), (Figure 2). During the same period, the proportion of preterm birth at our institution remained stable with slight upward trend (Figure 3). Our institution is the primary location for childbirth and features the only level III neonatal intensive care unit in the region.

### Respiratory support and PICU admission

Preterm children required higher level of support across all evaluated respiratory support modalities. Supplemental oxygen was used more frequently among preterm children than among term children (39% vs. 30.2%), as were HFNC (23.8% vs. 16.4%), CPAP/BPAP (13.0% vs. 8.6%) and mechanical ventilation (4.3% vs. 2.0%) (all *p* < 0.001). In adjusted logistic regression analysis, preterm birth was independently associated with higher odds of PICU admission (aOR 1.57, 95% CI 1.43 to1.72, *p* < 0.001) after controlling for viral pathogens, age and sex (Table 2). RSV and RV/EV were also independently associated with higher odds of PICU admission. Interaction analyses suggested that the association between preterm birth and PICU admission differed by viral pathogen, with evidence that RSV strengthened this association, whereas RV/EV appeared to attenuate it.

**Table 2:** Multivariable logistic regression analysis of factors associated with study outcome.

Variable	Adjusted OR (95% CI)	*P* value
Preterm birth	1.57 (1.43–1.72)	<0.001
RV/EV	1.66 (1.33–2.08)	<0.001
RSV	1.74 (1.32–2.30)	<0.001
Adenovirus	0.74 (0.54–1.01)	0.060
Influenza	0.45 (0.27–0.73)	0.001
hMPV	0.66 (0.37–1.18)	0.159
Parainfluenza	1.19 (0.82–1.76)	0.363
Age	NS	0.433
Sex	NS	0.375

OR, odds ratio; CI, confidence interval; RV/EV, rhinovirus/enterovirus; RSV, respiratory syncytial virus; hMPV, human metapneumovirus; NS, not statistically significant.

**Figure 4:**
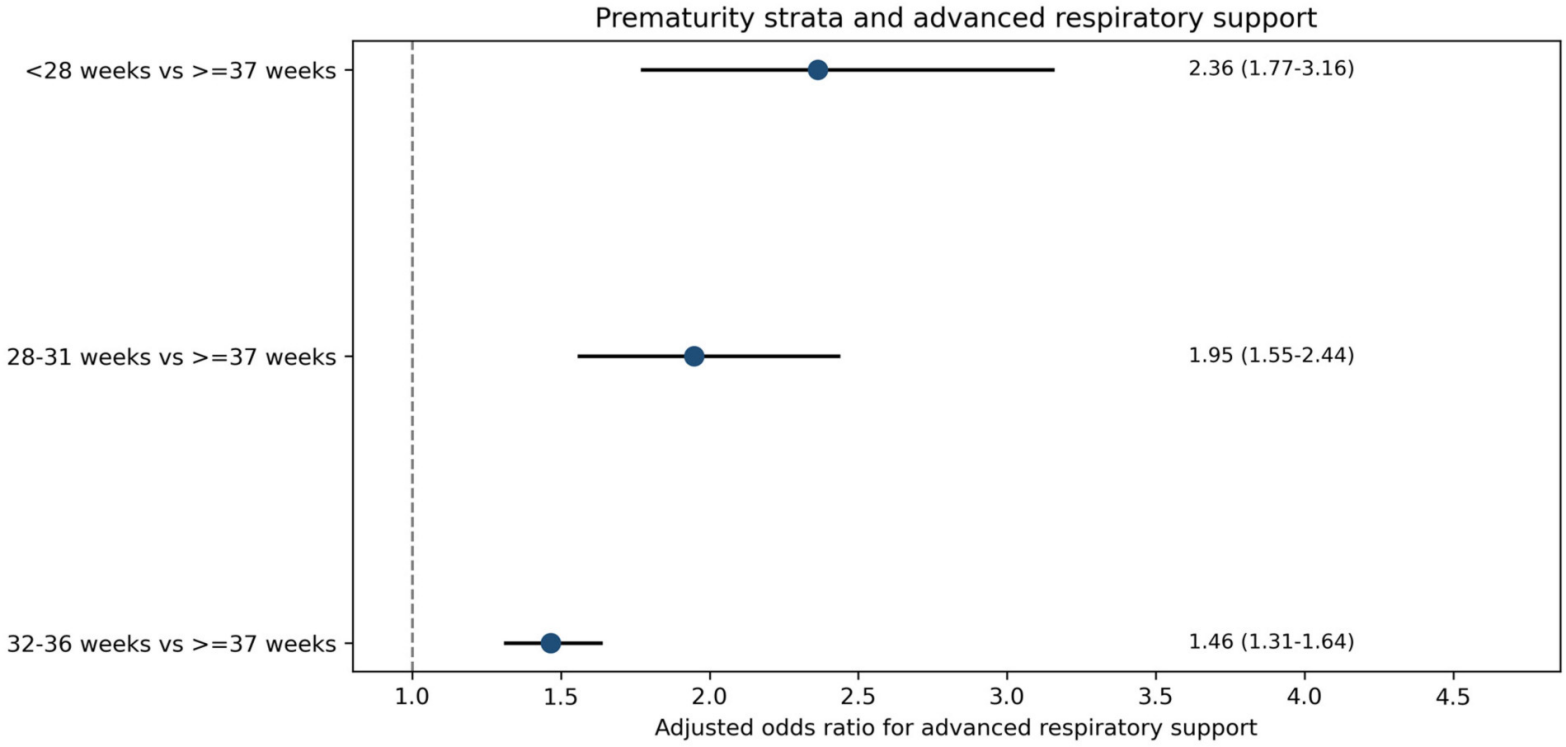
Adjusted odds of advanced respiratory support by prematurity strata. Forest plot showing the adjusted odds ratio (aOR) for advanced respiratory support in children born preterm at < 28 weeks, 28–31 weeks, and 32-36 weeks, using children who born at term (> = 37 weeks) as reference group. Lower gestational age was associated with greater odds of the need for advanced respiratory support. The dark circles represent the point estimates and the horizontal bars on either side of the dot represent the 95% confidence interval. The vertical dashed line represents the null value. Odds ratios were adjusted for age, sex and encounter year.

In gestational-age-stratified analyses using term infants (≥ 37 weeks) as the reference group, the adjusted odds (aOR) of advanced respiratory support increased progressively with decreasing GA, consistent with a dose-response relationship (Figure 4). Compared with term infants, aOR were 1.46 (95% CI 1.31 to 1.64) for children born at 32–36 weeks of gestation, 1.95 (95% CI 1.55 to 2.44) for those born at 28–32 weeks of gestation, and 2.36 (95% CI 1.77 to 3.16) for those born at <28 weeks of gestation.

In sensitivity analyses including prior encounters as a proxy for prior disease burden, prematurity remained independently associated with advanced respiratory support. Preterm birth was associated with aOR of 1.38 (95% CI 1.25 to 1.53), while prior encounters were also independently associated with greater severity, with an aOR of 1.15 per prior encounter (95% CI 1.13 to 1.17).

In pathogen-specific analyses, the association with prematurity and advanced respiratory support differed by the viral etiology (Table 3). The strongest associations were observed for RSV (aOR 2.31, 95% CI 1.72 to 3.11) and RV/EV (aOR 1.40, 95% CI 1.21 to 1.63), whereas corresponding associations were not statistically significant for influenza, adenovirus, hMPV, or sCOVs.

**Table 3:** Sensitivity analyses and pathogen-specific multivariable regression analyses.

Analysis	Comparison	Adjusted OR (95% CI)	*P* value	*N*	Events
Pathogen-specific analyses	RSV	2.31 (1.72–3.11)	<0.001	1,771	725
Pathogen-specific analyses	RV/EV	1.40 (1.21–1.63)	<0.001	6,538	1,727
Pathogen-specific analyses	Adenovirus	1.27 (0.74–2.18)	0.384	885	135
Pathogen-specific analyses	Influenza	0.98 (0.43–2.23)	0.967	805	69
Pathogen-specific analyses	HMPV	0.86 (0.48–1.52)	0.600	448	123
Pathogen-specific analyses	Coronavirus	1.19 (0.50–2.85)	0.693	449	45
Sensitivity analysis	Preterm vs. term	1.38 (1.25–1.53)	<0.001	17,045	3,629
Sensitivity analysis	Per prior encounter	1.15 (1.13–1.17)	<0.001	17,045	3,629
Gestational age strata	28–31 weeks vs. ≥37 weeks	1.95 (1.55–2.44)	<0.001	17,045	3,629
Gestational age strata	32–36 weeks vs. ≥37 weeks	1.46 (1.31–1.64)	<0.001	17,045	3,629
Gestational age strata	<28 weeks vs. ≥37 weeks	2.36 (1.77–3.16)	<0.001	17,045	3,629

OR, odds ratio; CI, confidence interval; RSV, respiratory syncytial virus; RV/EV, rhinovirus/enterovirus; HMPV, human metapneumovirus.

### Hospital LOS and mortality

Preterm birth was also associated with longer hospital LOS. In adjusted analysis, preterm birth was associated with an approximately 17% longer hospital LOS (beta = 0.16; 95% CI 0.09 to 0.23; *p* < 0.001). Mortality was rare (0.09%) and did not differ between groups.

No significant interaction was observed for mechanical ventilation, indicating that preterm birth was independently associated with higher odds of the need for mechanical ventilation regardless of the viral pathogen. Infection with the influenza virus was associated with a statistically significant lower odd for PICU admission (Table 2). In adjusted analysis, coinfection was associated with lower odds of PICU admission (aOR 0.83, 95% CI 0.45–0.85) suggesting that the presence of multiple viruses during the same encounter was not associated with higher illness severity.

## Discussion

In this large cohort study spanning seven years, preterm birth was independently associated greater severity of a pathogen-confirmed AVRI, in children presenting to the ED. Children with a history of preterm birth were more likely to require support across the full spectrum of respiratory support interventions, including supplemental oxygen, HFNC, CPAP/BPAP, and invasive mechanical ventilation. Preterm birth was also associated with increased odds of PICU admission and longer hospital LOS. These findings support the concept that preterm birth remains an important marker of host vulnerability in pediatric AVRI. Based on the chronology of lung development, this association is biologically plausible and likely reflects the effects of disruption in lung development associated with preterm birth, reduced pulmonary reserve, and alterations in immune function since most of the immunological benefits are conferred upon the fetus in the last trimester (12–14). Notably, the observed effect of preterm birth persisted after adjustment for age, sex, year, and viral pathogen, suggesting that preterm birth contributes to illness severity beyond the identity of the infecting virus alone (15, 16).

Additional analyses further suggested there was a gestational age gradient with progressively higher adjusted odds of advanced respiratory support with declining gestational age, rather than a uniform association with prematurity. This observation supports the concept that the degree of prematurity carries important prognostic information beyond a binary classification of term vs. preterm (16, 17). Our results also demonstrated that the association between prematurity and advanced respiratory support persisted after adjusting for prior encounters, suggesting that the increased severity observed among children with history of prematurity was not fully explained by prior health care utilization or recurrent respiratory morbidity.

In pathogen-specific analyses, the strongest association between advanced respiratory support were observed with RSV and RV/EV infection, raising the possibility that the impact of prematurity may be pathogen-dependent. However, these findings should be interpreted with caution, particularly with less common viral pathogen, but the findings further support the value of considering gestational age more granularly in risk stratification for children with acute respiratory illness evaluated in the ED.

An additional finding was the high frequency of RV/EV in this cohort, exceeding RSV as the most detected pathogen. This is consistent with the increasingly recognized role of RV/EV in clinically significant pediatric AVRI, particularly during and after the COVID-19 pandemic period. RSV and RV/EV were both associated with PICU admission, but the interaction analysis suggests that the relationship between preterm birth and severity may not be uniform across viruses. Specifically, preterm birth appeared to have a stronger association with PICU admission in the setting of RSV, whereas the association was less pronounced in the setting of RV/EV infection. Since this is an observational analysis, these findings should be interpreted cautiously (17, 18) and viewed as hypothesis-generating.

Importantly, while the interaction between prematurity and PICU admission was significant, no interaction was observed for invasive mechanical ventilation, suggesting that, while viral pathogen characteristics may influence intermediate levels of care, the risk of the most critical aspects of acute respiratory failure such as the need for invasive mechanical ventilation are driven largely by the underlying host vulnerability in the setting of AVRI.

Another notable finding in this study was the decline in the proportion of children with history of preterm birth over the period of the study. The number of preterm births during the same period (2019 to 2025) did not change significantly at our institution. However, this pattern may reflect the composition of ED encounters in this tested cohort rather than the incidence of respiratory illness among all children with history of preterm birth. Changes in the pattern of circulation of viruses, testing patterns by the clinicians, health-care seeking behaviors and nonpharmacologic mitigation measures during and after the COVID-19 pandemic may all have contributed to these observations.

The strengths of this study include the large ED-based cohort that spans multiple respiratory viral seasons, use of molecular diagnostic to identify multiple viral pathogens simultaneously during the same encounter and clearly defining outcomes that are clinically meaningful to ED, hospital and critical care practice.

This study also has several limitations. As a single center retrospective study our findings may not be generalizable to other clinical setting with different patient populations, practice patterns, and viral circulation patterns. The retrospective nature of the study also has the potential for misclassification, missing data, and residual confounding. The operational definition of AVRI identifies clinically significant viral respiratory infection presentation but may not distinguish lower respiratory tract disease from the boarder spectrum of viral respiratory illness with complete precision. The variability in ages of the children analyzed is another limitation of this study, since the impact of prematurity may manifest differently across infancy and childhood and we did have comprehensive data on atopic features in these children. PCR testing was at the discretions of the treating physician, and this may introduce selection bias. The practice for threshold for escalation of respiratory support may have varied over time across clinicians which can also introduce variations in the results. Additionally, if individual children contributed more than one encounter, within-patient correlation may not have been fully accounted for. And Finally, mortality was rare, limiting power for that outcome.

The findings of this study suggest that prematurity should be considered an important clinical risk factor when evaluating children who present to the ED with clinically significant AVRI. Futures studies should evaluate whether the association between prematurity and severity of AVRI differs by gestational age and weather addressing this issue helps with stratification for escalation of care or disposition.

## Conclusions

Among children presenting to the ED with PCR confirmed AVRI, preterm birth was associated with greater illness severity, including higher respiratory support requirements, greater likelihood of PICU admission, and longer hospital LOS. These findings supports that preterm birth is a clinically relevant marker of host vulnerability in clinically significant AVRI in children.

## Data Availability

The original contributions presented in the study are included in the article/Supplementary Material, further inquiries can be directed to the corresponding author.
