# Supplementary figures and images for: Association of preterm birth with severity of molecularly-confirmed acute viral respiratory illness presenting to the emergency department: a multi-year analysis
Source: Front Pediatr. 2026 Jun 2;14:1841637. doi: 10.3389/fped.2026.1841637 (PMC13269052; doi:10.3389/fped.2026.1841637)

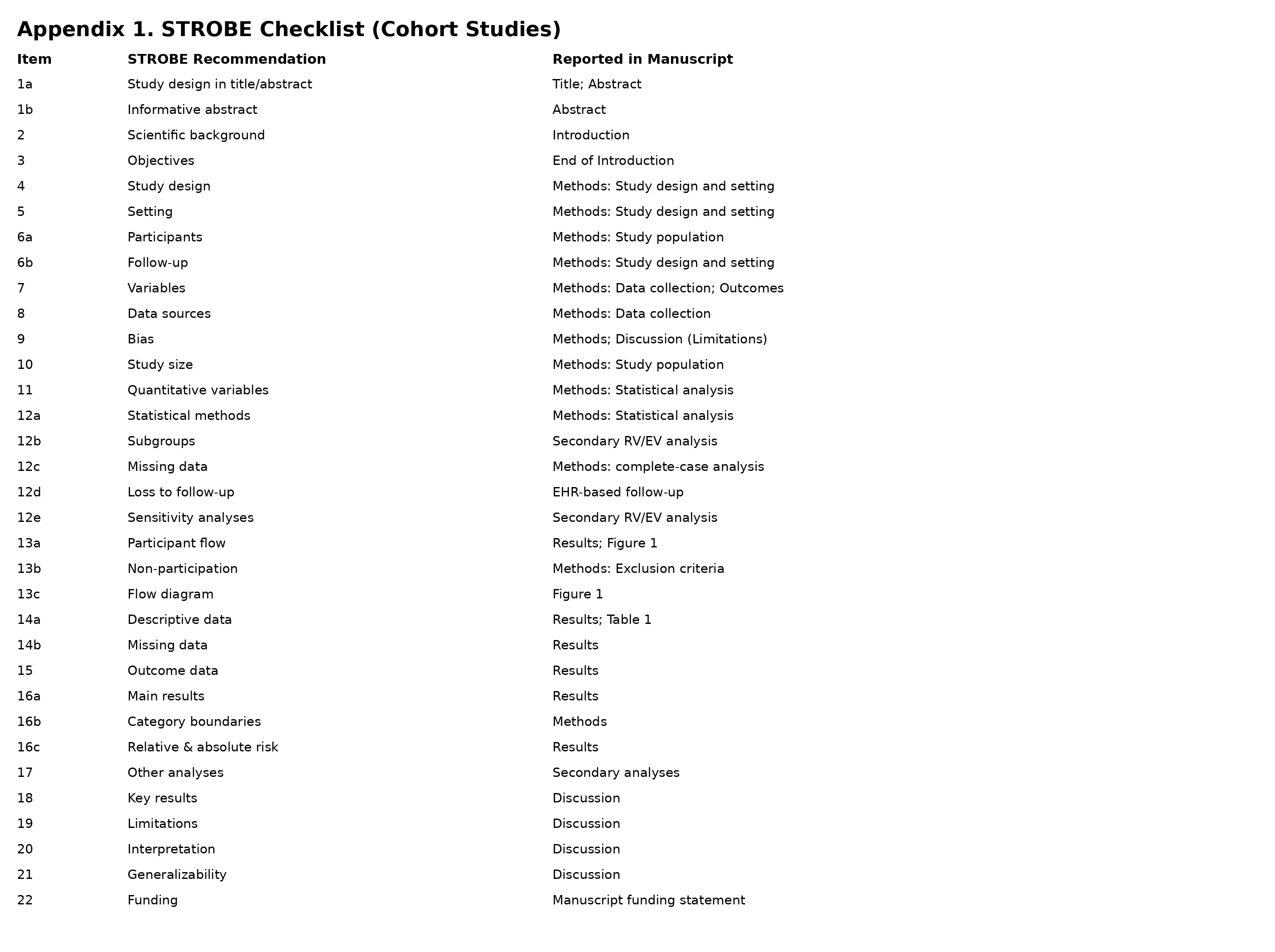

Supplement: Supplementary file 1 [file Supplementaryfile1.docx]
